# Covid-19 distance and online learning: a systematic literature review in pharmacy education

**DOI:** 10.1186/s12909-023-04346-6

**Published:** 2023-05-23

**Authors:** Muhaimin Muhaimin, Akhmad Habibi, Yasir Riady, Turki Mesfer Alqahtani, Anis Yohana Chaerunisaa, Tommy Tanu Wijaya, Tiana Milanda, Farrah Dina Yusop, Nour Awni Albelbisi

**Affiliations:** 1 Faculty of Pharmacy, Universitas Padjadjaran, Bandung, West Java, Indonesia; 2 Universitas Jambi, Jambi, Indonesia; 3 Universitas Terbuka, Banten, Indonesia; 4 Jazan University, Jazan, Saudi Arabia; 5 Beijing Normal University, Beijing, China; 6 Universiti Malaya, Kuala Lumpur, Malaysia

**Keywords:** Covid-19, Distance and online learning, Pharmacy Education, Systematic literature review

## Abstract

The Covid-19 outbreak necessitated the implementation of social distancing mechanisms, such as the enforcement of lockdowns in numerous nations. The lockdown has disrupted many parts of everyday life, but this unusual event has particularly affected education. The temporary closure of educational institutions ushered in dozens of new reforms, including a shift into the distance and online learning. This study investigates the transition from traditional education in physical classrooms to online and distance and online learning in pharmacy education during Covid-19, especially about the challenges and benefits of distance and online learning. We did Preferred Reporting Items for Systematic Reviews and Meta-Analyses (PRISMA) for literature sources between 2020 and 2022 (n.14). The study elaborates on how the transition has influenced teachers and students of pharmacy education. The research also summarizes several recommendations, which may assist in minimizing the adverse impacts of lockdown and encourage streamlined processes to distance and online learning, particularly in pharmacy education.

## Introduction

Covid-19, an infectious illness characterized by the SARS-CoV-2 virus, endangered the world quickly due to its highly infectious nature. As of 6 March 2023, there have been more than seven hudred million confirmed cases were documented, with over six million deaths [1]. The World Health Organization (WHO) labeled the virus a pandemic in March 2020. The pandemic caused havoc on a variety of activities of daily life, triggering governments worldwide to put in place a series of emergency response mechanisms [2, 3]. Country leaders imposed temporary closure and enforced extended isolation time, disrupting educational activity around the globe, reducing infection, and flattening the curve to avoid overburdening healthcare services. This resulted in the temporary closure of educational institutions in various parts of the world. The situation affected teachers, students, and their families [4–6]. Some academic institutions facing closure gradually reopened and began working under distance and online learning methods to keep students on track academically while also taking steps to mitigate the effects of the present health crisis. In the past, infectious disease epidemics have resulted in widespread school closures, with variable levels of success [7]. At the most basic level, distance learning refers to taking classes away from the college. Although technically a type of distance learning, online learning is more frequently used to describe programs where the instructors are not present simultaneously as the students [8].

Institutions have been forced into quickly transitioning to distance and online learning approaches mainly based on technology. Many educational stakeholders, such as teachers, students, and school administration staff, have not prepared to face the transition because of the fast switch to distance and online learning [9]. This transition to remote learning happened in an unexpected situation, leaving little time for teachers, educational staff, and students to prepare, modify, and adjust the learning. The condition brought several problems to the economy and social life. According to UNESCO, the temporary school closures enacted in response to the Covid-19 pandemic have impacted more than a billion students worldwide. Owing to a lockdown in 2020, students from over 50 nations have been kept out of school, accounting for roughly 18% of total registered students [10]. Many studies have been conducted to understand the impacts of distance and online learning in education due to Covid-19 [11–13]. However, literature reviews in a specific field of study are still limited and important to understand the broad effects of distance and online learning [7, 9, 14]. This systematic literature review takes an in-depth look at the studies on the influence of the Covid-19 pandemic on a specific field of education. This research examines how the shift from traditional methods to distance and online learning has affected teachers and students in pharmacy education. The impact of the pandemic-based distance and online learning on pharmacy education should be investigated to improve didactical decisions in the future and bridge the gaps to more adaptable but effective online pedagogical approaches. Initially, the focus areas of the literature review were investigated within pharmacy education. Following the focus areas, the distance and online learning challenges and benefits were assessed and elaborated on. Finally, recommendations of the prior studies included in this meta-analysis were concluded.

## Related work

Many governments were under pressure to prevent Covid-19 from spreading. This resulted in the temporary closures of many schools and universities [15]. Others switched to distance and online learning through technology. Viner et al. [16] did a systematic evaluation to determine the influence of school closures and other social distance techniques on disease rates and virus spread during crises. It was indicated that educational institution temporary closures play an insignificant role in virus transmission reduction. The minor advantages of such restrictions on the spread reduction might quickly be offset by the severe socio-economic implications [16–18]. The closure can have effects on individuals, families, and society. Therefore, any decision regarding school closures must carefully consider the potential trade-offs and aim to strike a balance between protecting public health and minimizing the adverse impacts on education, economy, and social well-being. As a result, many academic institutions have chosen the less drastic option of converting to distance and online learning [19, 20].

Distance learning refers to online instruction systems to create educational materials, provide teaching, and manage programs [21]. There are two basic types of distance learning: synchronous and asynchronous [22]. The main goal of distance and online learning is to replicate regular classroom communication approaches. Live webinars and virtual classes are examples of synchronous distance and online learning. On the other hand, asynchronous learning allows for greater flexibility in terms of timing which does not require real-time engagement; materials are provided online. Video recordings and emails are instances of asynchronous learning.

A comprehensive review and meta-analysis of controlled studies on the efficiency and approval of distance and online learning in medical sciences published between January 2000 and March 2020 evaluated students’ understanding, abilities, and satisfaction levels [23]. The study reported insignificant differences between traditional and distance and online learning regarding usefulness and objective assessments. Distance and online learning obtained a better approval rating in subjective assessments, suggesting that it was preferred to some degree by learners [23]. Carrillo & Flores [24] also reviewed the literature on online teaching and learning practices in teacher development between January 2000 and April 2020 to investigate online learning in teacher development and explain its consequences in the sense of the disease outbreak. The review discussed sociological, intellectual, and pedagogical problems and a comprehensive representation of innovation utilized to enhance teaching and learning [24].

Daoud et al. [25] performed a comprehensive review that evaluated the academic benefits of providing internet access at home, focusing on equality surrounding household internet access. It discovered several favorable associations between household internet access and the value of education for qualification, personal character, and social life. However, the relationship was not apparent and did not prove causality. Variables affect the aspects of online behaviors, including how technology is integrated and determine the educational value of household internet use [25]. Di Pietro et al. [26] published a report in which they attempted to investigate the consequences of the Covid-19 pandemic on education. It generated projections regarding the influence and future of learning based on pre and during Covid-19 data. The following are the four critical conclusions drawn from the article: (a) learning is likely to experience a stumbling block; (b) the impact on student achievements is likely to differ with economic factors; (c) social-economic disparity expressed in extreme reactions, less-wealthy families are subjected to greater environmental strain; (d) the broadening social inequality could have long-lasting effects [26].

Some virtual cases of emergency learning methods have been chastised for failing to follow basic pedagogical principles and guidelines [27]. Several studies have raised concerns regarding the possible negative consequences of rushing to introduce educational technology changes without first assessing their impact [27, 28]. Furthermore, the move to online education and distance and online learning technologies has sparked worries about spying and security and influenced students’ lifestyles [29]. In this research context, selected studies were diverse, from quantitative to qualitatitve approaches [30]. Because this phenomenon is still new, there is a lack of reflection on the pandemic digital revolution’s direct impact on postsecondary learning and its benefits, drawbacks, and future consequences.

## Method

The current research, a systematic literature review, follows the principles outlined in the preferred reporting items for systematic review and meta-analysis (PRISMA) procedures [31, 32], which include five stages: search, screening, eligibility, initial inclusion, and inclusion. PRISMA is a standard approach to assist researchers in transparently informing the study, steps, and results within the context of the systematic literature review [33].

### Research questions

This research investigates the effects of Covid-19 distance and online learning on pharmacy education. Four research questions were proposed: (1) what are the focus areas of the literature review? (2) what are the challenges of distance and online learning in pharmacy education? (3) what are the benefits of distance and online learning pharmacy education? (4) what recommendations were made?

### Search

The research questions were a basic guideline for determining the most popular search terms. The search includes terms synonymous with or closely linked to the main search phrases. The search was conducted using Science Direct, supported by Google Scholar search. The relevant search terms were used: “Distance learning in pharmacy education Covid 19,” “online education in pharmacy education Covid 19,” and “Technology integration in pharmacy education Covid-19.” The findings varied depending on the phrase combinations. However, in general, 17 to 81 papers (Table 1) were obtained each search, with the number growing relevant to the topics. Related phrases were gathered in all publications depending on the search. The terms sued in the search were determined through an in-depth discussion among the authors. We limited the terms so that future researchers can adapt this study for further investigation. The search limit provides narrow results for effective and efficient work for the most relevant answers to the research problems [34].

**Table 1:** Search in Science Direct (n. 137)

Keywords	Year	n.	Article type	n.
Distance learning in “pharmacy education” covid 19 (n.37)	2022	10	Research articles	20
	2021	22	Review articles	3
	2020	5	Book chapters	2
			Encyclopedia	2
			Data articles	3
			Discussion	4
			Short Communication	2
			Others	1
online education in “pharmacy education” covid 19 (n.81)	2022	24	Research articles	38
	2021	50	Review articles	5
	2020	7	Book chapters	2
			Encyclopedia	6
			Correspondence	2
			Discussion	9
			Case reports	2
			Others	17
Technology integration in “pharmacy education” covid-19 (n.19)	2022	4	Research articles	8
	2021	14	Review articles	2
	2020	1	Book chapters	1
			Encyclopedia	1
			Conference abstracts	1
			Others	6

Articles published after 2020 were kept in the study. Only works from high-quality journals were included; we selected the articles from indexed journals in Web of Science or Scopus databases. We initially reviewed the selected papers against Elsevier’s abstract and reference repository, Scopus, to verify that they were of top standard and didn’t relate to fraudulent publications. We also double-checked that they were in the Scopus indexation for the SJR, a measure of academic journals’ scientific impact. Furthermore, the publications were evaluated using Beall’s List, a list comprising predatory accessible publications that do not conduct an adequate review process.

### PRISMA procedures

**Fig. 1:**
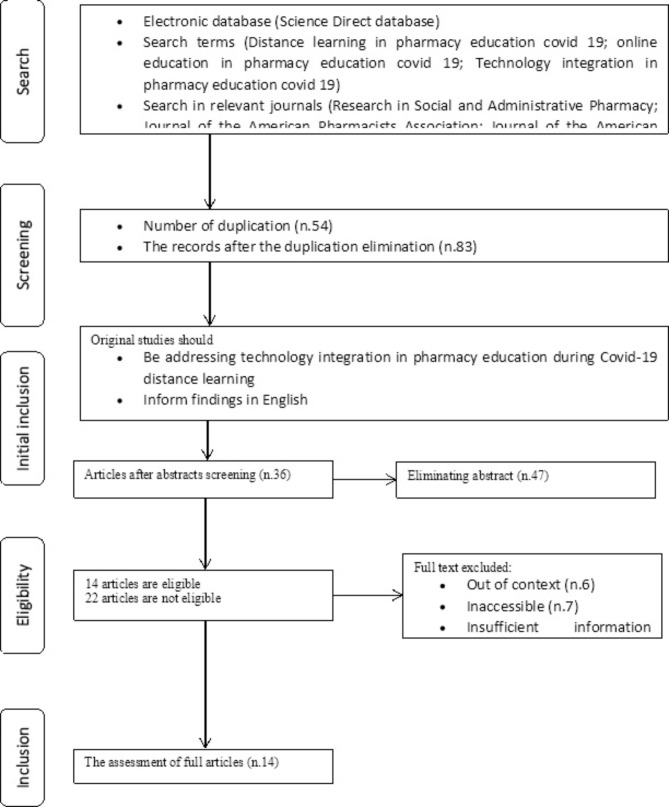
PRISMA flow diagram of the study

A reference list of scholarly papers directly referencing Covid-19 online learning: A comprehensive literature review in pharmacy education was created after merging these lists. After the first search or first phase, 137 scholarly publications were presented (Table 1). By removing duplicated results, we were able to screen for them. Microsoft Word was used as a tool in the duplication removal procedure. We went through each repeated title and removed them one by one. The redundancy led to 54 academic papers being sent for additional review, with 83 being deleted. Further, the step included the following elimination process; the articles should address technology integration in pharmacy education during Covid-19 distance learning, inform findings in English, be empirical studies (research articles), and be published from 2020 to April 1st, 2022. From the process, 47 abstracts were dropped, and the remaining 36 articles were for eligibility and inclusion (Fig. 1).

Following the initial screening, a review-coding method was performed using Macros in Microsoft Words for the abstracts, with the process documented by writing “included” 1st initial inclusion in the review box. After that, we added some information for every abstract, and the coding was done in a new draft where all the initial abstracts were included. The Macros [35, 36] were used to encode and extract the selected papers [37]. Macros were chosen because of their efficiency and functionality [35]. Tables were created to manage the comments and metadata. The study’s aim, method, study site/ population, and findings are listed in the tables. Four authors discussed and did the coding and combined documents into one before extracting the comments for analysis. The Macros were obtained for free at http://www.thedoctools.com/index.php?show=mt_comments_extract.

In the end, 14 articles were collected, examined, and reviewed. The criteria for inclusion in this systematic review were accessible articles in the context of distance education during Covid-19 in the field of pharmacy education. Meanwhile, the exclusion criteria included articles that were not in the context of Covid-19 (n.6), pharmacy education, and distance and online learning. Besides, inaccessible articles (n.7) and articles with insufficient information (n.9) regarding the topic were also excluded.

## Results and discussion

The results of this literature review are presented. The findings of every research topic are examined in depth. The focuse of the reviewed articles is presented in Table 2.

**Table 2:** Themes

Focus	Sources
*Influence on pharmacy Education*: Examines the impact of the shift from conventional teaching methods to more technology-based education.	Alqurshi [38]; Phillips et al. [39], Attarabeen et al. [40]; Montepara et al. [41], Ali et al. [42]; Alzubaidi et al. [43]
*Student Engagement*: analyzed the influence of the shutdown on individuals’ educational and behavioral lives and the distance and online learning experiences and academic achievement via online learning modalities.	Hamza et al. [44]; Alzubaidi et al. [43]; Higbea et al. [45]; Altwaijry et al. [46]; Stone and Pate [47]; Elbeshbeshy et al. [48]
*Regulation*: examines how people have responded to the outbreak and how strategy can help level the playing field in education.	Morgan et al. [49]; Reynolds et al. [50]; Etando et al. [51]

In the selected investigations, most educational institutions moved to online learning. The quality requirements listed in Table 3 were used to construct 14 studies.

### Area of focus

In this study, 14 publications considered the effect of COVID-19 on pharmacy education, specifically the technological change they sparked, distance and online learning challenges and benefits, and the recommendations for future studies. Eight papers discussed students’ and faculty’s experiences with remote learning and the participants’ perspectives on its possible benefits and drawbacks. Besides, four publications provided remote learning solutions or tested the performance of a specific technology. Three articles discussed educational policies considering the pandemic and examined the new approach to teaching and learning activities. Two papers investigated how the closure and subsequent transformation to technology-based education compounded achievement gaps. The gaps were revealed between students from lower-income households who lacked internet access and devices and those from higher-income families with devices and easy access to the Internet.

**Table 3:** Included sources

No	Author	Aim	Method	Population/study site	Findings
1.	Alqurshi [38]	To investigate the effect emergency, remote teaching has had on pharmacy education in Saudi Arabia	Survey and interview	Pharmacy students in Saudi Arabia	Challenges • Delivery of complex concepts of topics • Students-students interaction • Students-teachers interaction • Lack of guidance
2.	Phillips et al. [39]	Faculty restructured the pharmacology course with an active learning, facilitator-led classroom pedagogy.	Experimental study	Pharmacology of the University of Kansas Medical Center, Kansas City, USA	Challenges • Decreased study time Beneits • Faculty satisfaction with the new method of study
3.	Ali et al. [42]	To Explore pharmacy students’ perspectives on its impact on their learning.	Qualitative; Twitter chat	Pharmacy students in Saudi Arabia	Benefits • Easier and frequent communication • Time efficiency Challanges • Lack of teachers’ knowledge about online learning • Lack of teachers’ experience • Limitation of technology • Rearrangement of course assessments Suggestions • The availability of recordings of ‘live’ lectures
4.	Etando et al. [51]	To understand the challenges and how these were addressed, given increasingly complex patients, to provide future direction.	Exploratory study	Senior-level medical and pharmacy educators across Africa	Challenges • Adapting to online learning. • Lack of equipment (especially among disadvantaged students), • The costs of internet bundles and how to conduct practical and clinical teaching. Recommendation • Training sessions, • Developing innovative approaches to teaching, and • Seeking ways to reduce internet costs
5.	Alzubaidi et al. [43]	To explore pharmacy colleges’ experiences and challenges worldwide with the transition to online teaching during covid-19	Survey	111 pharmacy colleges from 28 countries	• The majority of faculty (75.0%) and administrators (61.9%) reported moderate work-related stress. • Most academics felt that they received adequate support from their institutions and had positive perceptions of the transition to distance e-learning during the pandemic.
6.	Altwaijry et al. [46]	To describe the experience of academic staff and students with distance education during the covid-19 pandemic	A mixed-method approach; survey and interview	Academic staff and students of the college of pharmacy in Saudi Arabia	• Positive for readiness for the shift to distance education during the full lockdown • Positive perception toward distance education • Distance education is perceived positively
7.	Montepara et al. [41]	To implement a health system of collaborative learning	Experimental study	Thirteen pharmacy schools in the USA	• Positive experience with distance and online learning
8.	Higbea et al. [45]	To highlight gaps or potential pitfalls in preparing students to enter the healthcare field.	Observation study	Six colleges and schools of pharmacy: Auburn University, Drake University, Purdue University, University of Health Sciences and Pharmacy in St. Louis, Texas Tech University Health Science Center, and University of Utah	These lessons learned to pertain to remote • Content delivery, • Student engagement, • Skill development and assessments Recommendations • Lecture-based learning, • Group-based learning, • Lab-based learning, • Assessments • Training learners for remote patient care and remote experiential activities and assessment. • Future research for remote learning within pharmacy education
9.	Hamza et al. [44]	To assess pharmacy students’ knowledge, attitudes, and practices towards the Covid-19.	Survey	Senior pharmacy students of Egypt	Challenges • Lack of information • Lack of knowledge of major causes of death in patients Benefits • It was controlled successfully. • Sufficient practice toward Covid-19 Recommendation • More attempts to protect pharmacists.
10.	Reynolds et al. [50]	To deliver initial practical professional skills on Covid-19	Survey	University of Colorado’s International-Trained PharmD students	Benefits • Successful online course design • Improvements in knowledge and skills
11.	Morgan et al. [49]	To examine how a school of pharmacy creatively approached the challenge of online assessment while maintaining the standards necessary to prepare practice-ready student pharmacists	Survey	Schools of pharmacy located at two campuses in Virginia, USA	Benefits • Online video proctoring maintained consistency in exam structure and administration Challenges • Students preferred unproctored, open-book, internet access-enabled, standard time exams versus proctored Recommendation • Changes to testing procedures, whether with proctored or unproctored methods, appeared to increase student stress.
12.	Attarabeen et al. [40]	This research aimed to investigate whether there was an increase in student-perceived stress due to the Covid-19 pandemic	Survey	Pharmacy students in a public pharmacy school, USA	Challenges • Coping behavior • Emotional status • Self-efficacy
13.	Elbeshbeshy et al. [48]	To elaborate on the self-reported impact of the Covid-19 pandemic among final-year students in pharmacy education	Mixed method (survey and interview)	Pharmacy students in New York, USA	Challenges • Covid-19 negative impact on students’ future career • Perspective change • Experience gaining • Positive outlook.
14.	Stone and Pate [47]	To discusses the impact of COVID-19 on pharmacy students	Experiential study	A 4th-Year Pharmacy Student, University of Mississippi, School of Pharmacy, Oxford, Mississippi	Benefits • Strengthened the resilience of the students. • Continued advocacy for the profession and articulation • Inspired students in raising awareness of Covid-19 • Uncertainty over future

### Challenges

The key challenges can be summarized in the following points: *disparity in accessibility, training insufficiency*, *lack of communication, technical issues, pressure, work, and confidence, and lack of student involvement, technical knowledge, and performance evaluatio*n.

There is a *disparity in accessibility* for pharmacy students, typically linked to family income [42, 45, 46, 51], discussed in four articles from the review sources. The shift to distance and online learning worsened the disparities between wealthy and disadvantaged pharmacy students. Students studying pharmacy in less affluent areas have little or no access to supporting devices and the Internet [42, 45]. Students from low-income families were reported to have less skill and knowledge of technology than students from high-income families with strong economic backgrounds [38, 41]. The inequality goes to institutions located in rural areas, which are under-equipped compared to institutions located in cities or urban areas [52], resulting in different challenges faced by each type of institution.

While technology can enhance the learning experience, it cannot completely replace it, especially in pharmacy professions requiring hands-on laboratory training that indeed produces *training insufficiency* [44, 48]. The phenomenon is especially true in health-related fields, such as pharmacy. The papers on pharmacy education emphasized the importance of hands-on experience and how secondary knowledge derived through simulation, presentation recordings, or online meetings through video conferencing cannot replace the experience.

Because of the depreciation or lack of physical interaction and the intrinsic vagueness of textual exchanges, forming and maintaining connections and forging communication between students, their classmates, and their teachers became increasingly challenging [38]. With the inexistence of visible touch and the capacity to observe students in classrooms, teachers and instructors have a more challenging time explaining directions and evaluating student response, involvement, and participation. These *lack of communication* challenges have been revealed in three articles within this literature review [38, 40, 45].

*Technical issues* such as Internet or Wi-Fi access, tool malfunctions, and stream stability might obstruct communication [42, 45, 51]. As the pandemic spread over the globe, accessibility to a dependable internet connection became increasingly vital in the last year, and quite enough of day-to-day life shifted from in-person to online. Many students, however, have suffered from technological challenges since the start of Covid 19, and existing disparities have indeed been exacerbated by the lack of consistent accessibility [42, 45, 51].

*Pressure, work, and confidence* were all impacted by the students’ and teachers’ forced and quick transfer to remote learning. Many pharmacy students and faculty members faced financial and social anxiety due to the lockdown, which indirectly impacted their performance. Academic employees, for example, had to deal with increased or even quadrupled workloads. Extended time without face-to-face social interaction can also harm one’s mental health.

*Technical knowledge* is the next challenge of the current study [42, 45, 46, 51]. Many educational institutions, schools, and universities were surprised by this rapid and forced digital change, giving educational leaders limited time to educate their professional personnel. The complex evidence and reality left non-tech-aware teachers and instructors unprepared and unequipped to work with complex technological-based activities. Teachers’ lack of technical expertise and prior experience using online tools are also challenges [42, 51]. In many circumstances, the incapacity of faculty members to use technology hampered the success of distance and online learning.

Other difficulties include a *lack of student involvement and performance evaluatio*n [40–42, 45, 51]. Student engagement was occasionally weak due to dependency on recorded meetings, limitation of intention, and stress produced by using the devices. There was also weariness from staring at screens for long periods, isolated thoughts, and melancholy from a limited personal touch [40, 42]. Teachers faced problems revising learning assessments to fairly record student academic performance and achievements [51], which is challenging during distance and online learning, especially for pharmacy students.

Other challenges might also be faced during distance and online learning due to Covid-19. The quality of online and distance learning in pharmacy education is one of them, and it can be a major issue. The government’s educational policy makes no explicit mention of distance and learning. Lack of quality control, development of e-resources, and content delivery can be present. This issue needs to be addressed in further work, especially in pharmacy education, so that all stakeholders can take advantage of the advantages of high-quality distance and online education. One should consider developing and improving the quality of learning for future pandemics.

### Benefits

This stage highlights the benefits of digital change in pharmacy education for more opportunities in the future of education. There are a number of benefits [39, 41, 42, 45, 47, 49, 50] informed by sources included in this systematic literature review, namely *bridging the gap between time and place, communication effectiveness, information transition, and cost-effectiveness.*

Distance and online learning *bridge the gap between time and place*, that gives pharmacy students and teachers the freedom to listen to academic lectures and speeches from the coziness of their living rooms or from anywhere else [42, 47, 50]. Due to the time, it also enables pupils to self-regulate their education and progress at their own pace. Distance and online learning give students the opportunities to listen to their lectures from the comfort of their own homes or from anywhere else. Because of the adaptability enabled by elements such as recording, distance and online learning also helps students to self-regulate their learning and continue at their speed. Online learning allows for a more modern and practical way of communication [39, 41, 47, 49, 50]. Significant debates might be addressed during courses, and participants can profit from these talks by observing or engaging in chat.

Distance and online learning facilitate *communication effectiveness* because participants shouldn’t have to talk face to face or deal with the anxiety that comes with talking in front of a live audience, which encourages more conversation. Parents of young children can also benefit from online learning by becoming more active in their children’s education [39, 45, 47, 49]. The pressures of the pandemic to shift to digital and remote educational models in teaching revealed flaws in the approach and compelled lecturers to consider and evaluate present and prior instructional approaches, offering a glimpse into what educational technology could look like, encouraging didactical advancement and accelerating changes in technology-based education. The process can be considered a catalyst for curricular and classroom improvement [39, 49, 50].

The employment of simulations and other approaches for educational goals and the deployment of online learning are seen as beneficial and adequate, if not comprehensive, substitutes for traditional learning [39, 41, 42, 45, 47, 49, 50]. It met the goal of continuing to provide instruction in the face of the epidemic while also assisting pupils in meeting their expectations. Distance and online learning also help increase *information transmission*, with additional benefits of *cost-effectiveness*. Students are exposed to new and relevant technologies by integrating technology into education [39, 45, 49].

### Recommendations and suggestions

The solution is raising and sustaining their motivation to promote morale and battle any lockdown-induced stress or worry. Accessible online learning portals are for institutions in pharmacy education. Generating and accepting feedback from learners to ensure the quality of online learning is another piece of advice made by the existing literature in pharmacy education [39, 42, 43, 45, 49]. They are examining the outcomes of distance and online learning and commenting on the distinctions between it and traditional education to identify which components are sustainable and fit the expectations placed on pharmacy education in general by the pandemic situation.

The current study also helps lecturers use effective instructional strategies and allows educational institutions to enhance online instructional resources continuously [53, 54]. Pharmacy students comprehend the required courses and sense the connection of the study content to the actual world. Teachers must set clear expectations and establish course objectives and the value of the syllabus to accomplish this [39, 49]. Early in the academic year, they must also define their roles and duties as instructors and facilitators [43, 45]. Furthermore, authorities should aim to assess and prevent any dangers or disadvantages of economic or workload discrepancies because of this rapid transition from traditional learning to distance and online learning during crises like Covid-19 [55].

Another piece of advice is to reassess and rethink educational practices and formulate guidance to steer the shifts to online and distance learning and make necessary infrastructural improvements [56, 57]. The activities are designed to familiarize students and professors with technology, develop their competence, and equip them to deal with technological challenges that may arise during online lectures [49]. This will also aid in the effective use of technology to fulfill its full potential in online education. Finally, it is critical to provide underequipped pupils with the essential tools to participate in online communications, such as devices and solid internet access [39, 45].

## Conclusion and future work

Covid-19 has a major effect on the world and how people arrange themselves in the actual world. It has revealed systemic flaws inside institutions and resulted in lengthy changes. This was also true in the educational system. This assessment aimed to examine and assess the impact of these developments on pharmacy education. In total, 14 articles regarding distance and online learning during Covid-19 were discussed. The current study uses the PRISMA approach to outline the findings through 5 steps (search, screening, eligibility, initial inclusion, and inclusion). To fill the gap of prior studies in pharmacy education, we examined the change in learning from traditional methods to distance and online learning, affecting all related stakeholders. The impact of pandemics on pharmacy education should be more elaborated for future research for the betterment of education, especially pharmacy education. In short, we focus the presentation of the study on the focus areas of the literature, benefits, and challenges of distance and online learning during Covid-19 in pharmacy education.

## Data Availability

The datasets used and/or analysed during the current study available from the corresponding author on reasonable request.
